# Evaluation of Dewatering Performance and Fractal Characteristics of Alum Sludge

**DOI:** 10.1371/journal.pone.0130683

**Published:** 2015-06-29

**Authors:** Yongjun Sun, Wei Fan, Huaili Zheng, Yuxin Zhang, Fengting Li, Wei Chen

**Affiliations:** 1 Key laboratory of the Three Gorges Reservoir Region's Eco-Environment, State Ministry of Education, Chongqing University, Chongqing, China; 2 National Centre for International Research of Low-carbon and Green Buildings, Chongqing University, Chongqing, China; 3 National Key Laboratory of Fundamental Science of Micro/Nano-Devices and System Technology, College of Materials Science and Engineering, Chongqing University, Chongqing, China; 4 Key Laboratory of Yangtze River Water Environment, Ministry of Education, State Key Laboratory of Pollution Control and Resource Reuse, College of Environmental Science and Engineering, Tongji University, Shanghai, China; Peking UIniversity, CHINA

## Abstract

The dewatering performance and fractal characteristics of alum sludge from a drinking-water treatment plant were investigated in this study. Variations in residual turbidity of supernatant, dry solid content (DS), specific resistance to filtration (SRF), floc size, fractal dimension, and zeta potential were analyzed. Sludge dewatering efficiency was evaluated by measuring both DS and SRF. Results showed that the optimum sludge dewatering efficiency was achieved at 16 mg∙L^-1^ flocculant dosage and pH 7. Under these conditions, the maximum DS was 54.6%, and the minimum SRF was 0.61 × 10^10^ m∙kg^-1^. Floc-size measurements demonstrated that high flocculant dosage significantly improved floc size. Correlation analysis further revealed a strong correlation between fractal dimension and floc size after flocculation. A strong correlation also existed between floc size and zeta potential, and flocculants with a higher cationic degree had a larger correlation coefficient between floc size and zeta potential. In the flocculation process, the main flocculation mechanisms involved adsorption bridging under an acidic condition, and a combination between charge neutralization and adsorption-bridging interaction under neutral and alkaline conditions.

## Introduction

All drinking-water treatment plants produce water treatment residuals during the process of water purification for human consumption. These residuals mainly consist of organic and inorganic compounds in solid, liquid, and gaseous forms, with variable compositions and different physical, chemical, and biological characteristics [1]. Water treatment residuals from drinking-water treatment plants are mainly sludge water in sedimentation tank and filter backwash. Suspended solids content is generally about 1000 mg∙L^-1^, sometimes even above 10000 mg∙L^-1^. The volume of water residuals depend upon the treatment technology involved in the water treatment process and the raw water turbidity. If the sludge water is not treated and is directly discharged into sanitary sewers or nearby surface water, it would cause sewer blockages or siltation of rivers and lakes.

Aluminum salts are usually used as coagulants in modern drinking-water treatment plants and are mostly concentrated in the sediment sludge. The alum in the sludge water generates a certain effect on aquatic plants, water microorganisms, and microbial wastewater treatment plant. A large number of previous research studies [2–6] have shown that the solubility of aluminum has certain toxic effects on the human body, animals, plants, and microorganisms; it induces toxicity to aquatic organisms and impairs the quality of the systems. Sotero-Santos [7] suggested that discharge of alum sludge can impair the quality of water and may have chronic effects on aquatic communities. Moreover, excess coagulants are deleterious to biological processes when the alum sludge water is discharged into municipal wastewater treatment plants through municipal sewage pipe network [8].

Depending upon the physical and chemical quality, these waste residuals can be utilized in agriculture and silviculture, brick manufacture, recovery of alum for reuse as a coagulant, and control of phosphorus and removal of heavy metals from wastewater [9–11]. Water treatment sludge should be flocculated and dewatered before final disposal and reuse to reduce transport cost and utilization efficiency. Although various alternative options for conditioning alum sludge have been suggested [12], the search for cost-effective and high dry solid content treatment process has become an urgent priority because of tight environmental regulations and increasing disposal costs.

The research and engineering practice about alum sludge treatment in China started later than in America and Europe. With the continuous development of China's environmental protection, sludge water treatment systems had been built and put into operation in Beijing, Shanghai, and other cities in recent years. With the development of industry and society, and the improvement of environmental protection requirements from the state environmental protection department, the environmental pollution caused by sludge water from drinking-water treatment plants has drawn more and more attention.

According to a reference [13], Fractal-like properties such as self-similarity can distinguish between fractals. The self-similarity was used interchangeably with scaling and scale invariant in literature, which simply means that the pieces of the object resemble the whole object. Therefore, fractal dimension reveals the characteristics of self-similarity. Microscopic measurements have been used to analyze the fractal dimension[14]. This method measures the average fractal dimension of flocs in the target regions. Jin [15] observed that the fractal dimension has a relatively strong effect on dewaterability; high fractal dimension values (1.96–2.44) were derived and calculated from raw light scattering data for activated sludge. Large fractal dimensions indicate dense flocs, which are desired in the flocculation process [16]. Among several other researches, image analysis method is applied to calculate the fractal dimension. Fractal dimension exhibits a linear relation between Logarithmic projected area and Logarithmic projection perimeter. There has only few studies investigating the fractal dimension of sludge flocs with respect to solid contents. Thus, determining whether or not a relationship exists between these factors in raw and flocculanted alum sludge matrices merits considerable interest [17].

In this study, the response of sludge dewatering performance to varied flocculation conditions was investigated, and the optimum polymer dose was gauged by dry solid content (DS) and specific resistance to filtration (SRF). Flocs property tests were conducted to identify the floc size and zeta potential after flocculation process. Floc morphology and fractal dimension were examined in great detail using image analysis method. Finally, the study focused on a discussion of the correlations between fractal dimension and floc physical properties.

## Materials and Methods

### Sludge

The sludge tested was sourced from water treatment plant in Chongqing (China). No specific permissions were required for these locations/activities. The water treatment plant was the model of environmental protection education. A large number of elementary school students and middle school students would visit the water treatment plant for study the knowledge of environmental protection and water treatment weekly. The field studies did not involve endangered or protected species

All experiments would be completed in 48 h after the sludge samples were obtained. The scale of water treatment plant was 20 × 10^4^ m^3^∙d^-1^, and the raw water was sourced from Jialing River with turbidity levels between 10 and 40 NTU currently. Polyaluminum chloride (PAC) was used as coagulant in the flocculation process and was dosed at 3 kg PAC per 1000 m^3^ raw water. CPAM was synthesized in our laboratory; the detailed synthesis and characterization of CPAM has been previously reported [18–20]. The characteristics of sludge from Jingkou water treatment plant are shown in Table 1, and the flocculants used for dewatering the sludge are shown in Table 2.

**Table 1:** Characteristics of sludge from Jingkou water treatment plant.

pH	Mass density (g∙mL^-1^)	Dry solid content (%)	Zeta potential (mv)	Al content (mg∙mL^-1^)
**7.0±0.1**	1.196	8.41	-9.04	220±10

**Table 2:** Flocculants used for dewatering sludge from water treatment plant.

Flocculant	Full name	Cationic degree (%)	Intrinsic viscosity (dL·g^-1^)
CPAM1	Poly(acrylamide-acryloyloxyethyl trimethyl ammonium chloride-butylacrylate)	38	1.70
CPAM 2	Poly(acrylamide-acryloyloxyethyl trimethyl ammonium chloride -butylacrylate)	40	2.38
CPAM 3	Poly(acrylamide-acryloyloxyethyl trimethyl ammonium chloride -butylacrylate)	21	2.41

### Flocculation and dewatering experiments

As described in detail previously [21–22], a program-controlled Jar-test apparatus (ZR4-6 Jar Tester, Zhongrun Water Industry Technology Development Co., Ltd., China) was used for sludge dewatering experiments. The dewaterabilities of the sludge flocculated with cationic polyacrylamide (CPAM) were assessed in terms of residual turbidity of the supernatant, DS, SRF, zeta potential, and floc size. Dewatering tests for alum sludge were carried out at room temperature. Each result was an average of three repeated tests under similar experimental conditions. The pH of the sludge system was adjusted by adding NaOH (1.0 mol·L^-1^) or HCl (1.0 mol·L^-1^). Alum sludge (500 mL) was transferred into beakers before the requisite amounts of flocculants (CPAM) were added. The sludge solution was rapidly mixed at 100 rpm for 30 s, slowly stirred at 30 rpm for 2 min, and sedimented for 10 min.

### Analytical method

The text reproduces(reproduced) information (that)had been already reported in details in our previous work [21–22]. Vacuum filtration was performed to measure the dewaterability of the flocculated sludge. The supernatant of the sludge after flocculation was extracted for turbidity measurements using a turbidimeter (HACH 2100Q, U.S.). After rapid agitation, the zeta potential of the supernatant was measured with Zetasizer Nano ZS90 (Malvern, U.K.). The flocculated alum sludge flocs were carefully gathered, and then separately set up for image recording with MIT300 metallurgic microscope (Chongqing OTT Optical Instrument Co., Ltd., China). Image-pro Plus 5.0 was used to determine the fractal dimensions. The fractal dimensions for the flocs were calculated by regression analysis of the logarithm of their projected areas versus the logarithm of their corresponding perimeters. In this study, the longest diameter of the aggregate image was considered as the characteristic length [23]. The microscope photos of flocs were shown in the S1 Fig and S2 Fig.

As described in detail previously [21–22]. Meanwhile, the sludge floc was measured simultaneously with BT-9300H laser particle size distribution instrument (Dandong Baite Technology Co., Ltd., China). The flocculated sludge was poured into a Buchner funnel for filtration at 0.015 MPa vacuum pressure for 3 min or until the vacuum can no longer be maintained for <5 min. The filterability of the sludge was measured by the specific resistance of the sludge. DS was calculated using the following equation:

$$\text{DS}=\frac{W_{2}}{W_{1}}\times 100\%$$ (1)

where *W*_1_ is the weight of wet filter cake after filtration and *W*_2_ is the weight of filter cake after drying at 105 °C for 24 h.

SRF was calculated using following equation [24–25]:

$$SRF=\frac{2bPA^{2}}{\mu c}$$ (2)

where *P* is the pressure of filtration (N/m^2^); *A* is the filtration area (m^2^); *μ* is the viscosity of the filtrate (N∙s/m^2^); *c* is the weight of solids per unit volume of filtrate [kg/m^3^, *c* = 1/*C*_i_/((100*C*_i_)–*C*_f_)/(100*C*_f_)), where *C*_i_ and *C*_f_ are the initial and final moisture content (%), respectively]; and *b* is the slope determined from *t*/*V*_f_(*y*)–*V*_f_(*x*) plot, where *V*_f_ is the volume of filtrate (m^3^) and *t* is the filtration time (s).

## Results and Discussion

### Effect of dosage on the sludge dewatering performance

**Fig 1:**
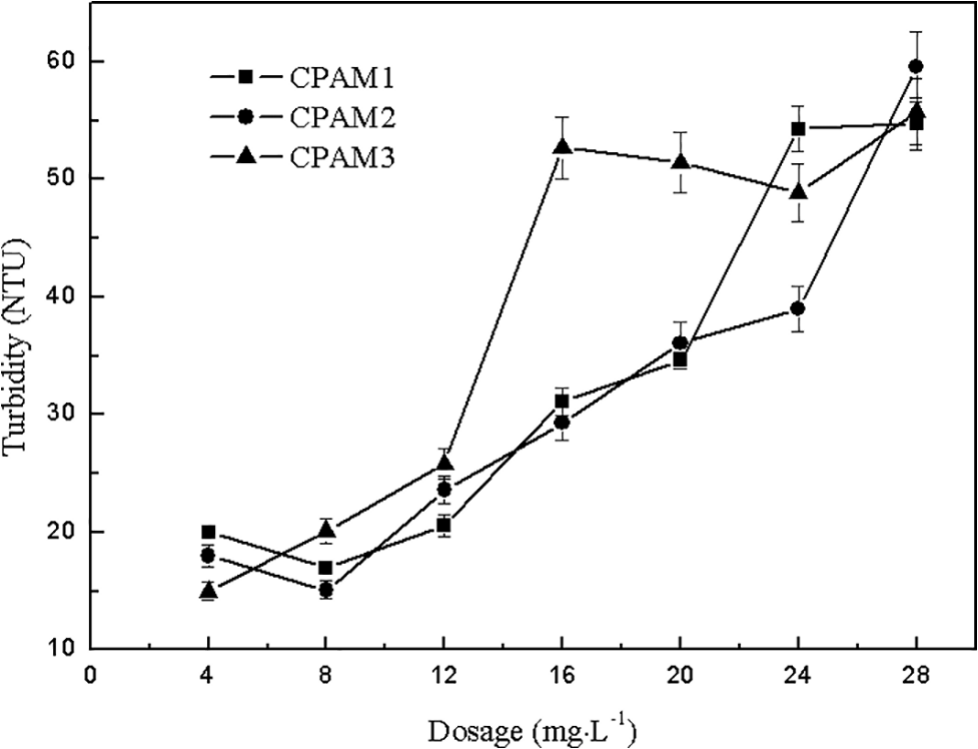
Effect of dosage on the residual turbidity of supernatant.

**Fig 2:**
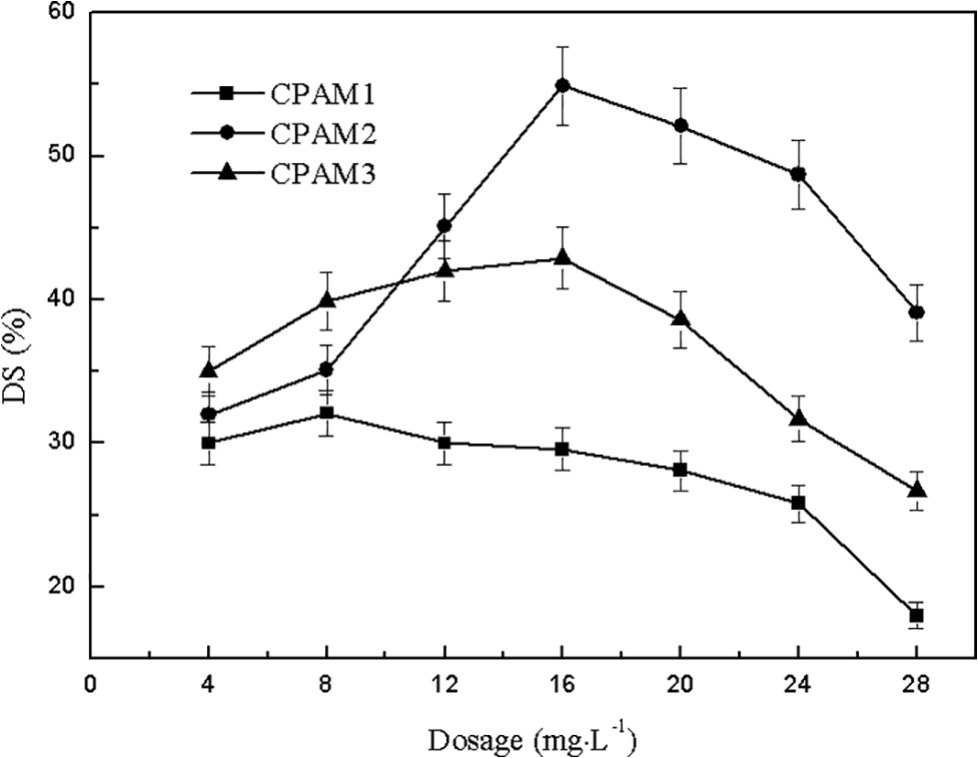
Effect of dosage on DS.

The effects of dosage on sludge dewatering performance are shown in Figs 1 and 2. With increased dosage, the residual turbidity of supernatant increased gradually. However, CPAM1 and CPAM2 showed an increase only after a weak decrease at 8 mg2219L^-1^, where minimum residual turbidities of 17.0 and 15.1 NTU, respectively, were obtained. These results may be because the drinking-water treatment system took PAC as coagulant, and the PAC that existed in the residual sludge was positively charged. Positively charged cationic flocculants interacted with PAC and produced electrostatic repulsion, leading to the escape of some small sludge particles from the flocs. With increased dosage of cationic flocculant, the electrostatic repulsion force strengthened, resulting in increased turbidity of the supernatant. Thus, increasing the coagulant dosage caused the turbidity of the supernatant to continue increasing. A comparison in Fig 1 shows that the supernatant turbidity of CPAM3 was higher than that of CPAM1 and CPAM2, indicating that a high cationic degree favors better flocculation performance.

As shown in Fig 2, with increased dosage, the DS of dewatered sludge increased initially, and then decreased. The maximum DS of CPAM1, CPAM2, and CPAM3 were 32.1%, 54.9%, and 42.8% obtained at 8, 16, and 16 mg∙L^-1^, respectively. When the added flocculant was greater than 12 mg∙L^-1^, the CPAMs in the order of decreasing DS values were as follows: CPAM2 > CPAM3 > CPAM1. The DS of CPAM2 was significantly higher than that of CPAM1 and CPAM3. A comparison between CPAM1 and CPAM2 suggested that CPAM with higher intrinsic viscosity results in a higher DS at the same cationic degree. The comparison between CPAM2 and CPAM3 indicated that with higher cationic degree at the same intrinsic viscosity, CPAM shows higher DS [26].

High cationic degree contributed to the efficient ability to neutralize negative charge, resulting in effective flocculation performance. The greater the intrinsic viscosity is, the longer are the flocculants’ molecular chains, which function in adsorption bridging in the sludge flocculation process. Under the same conditions, the flocculants with a high cationic degree or high intrinsic viscosity were conducive in improving and enhancing the performance of flocculation and sludge dewatering. Therefore, the optimum flocculant dosage for dewatering alum sludge ranges between 12 and 20 mg∙L^-1^, where 16 mg2219L^-1^ was considered as the optimum dosage.

### Effect of pH on sludge dewatering performance

**Fig 3:**
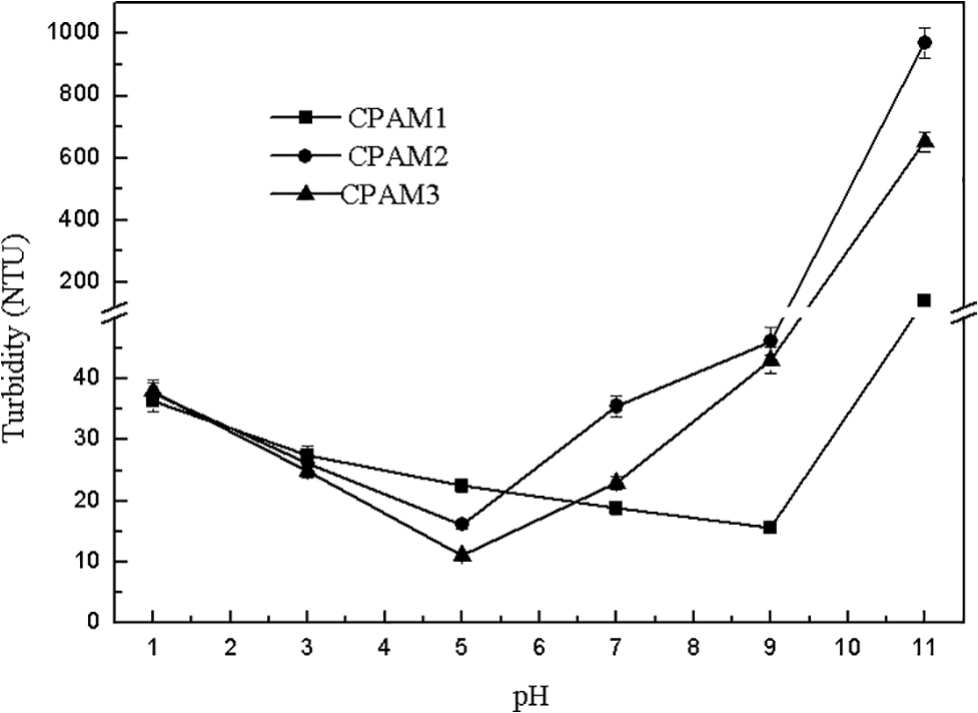
Effect of pH on the residual turbidity of supernatant.

Fig 3 shows the effect of pH on the residual turbidity of the supernatant after flocculation. The supernatant turbidity of CPAM1 decreased with increased pH. However, with increased pH, the supernatant turbidity of CPAM2 and CPAM3 decreased initially until pH 5, and then increased dramatically. The minimum residual turbidity of CPAM1 and CPAM2 supernatants were 16.1 and 11.2 NTU, respectively, obtained at pH 5. Meanwhile, the residual turbidity of CPAM1, CPAM2, and CPAM3 were also determined at pH 11, obtaining 143, 971, and 653 NTU, respectively, which were much larger than the values of residual turbidity obtained at other pH values. The high residual turbidity values obtained at pH 11 show that flocculation performance deteriorated significantly in strong alkaline conditions. During the flocculation tests, sludge flotation was observed at pH 1, and the supernatant was yellow. At pH 11, the supernatant, which is a mixture of many fine sludge particles, was muddy.

**Fig 4:**
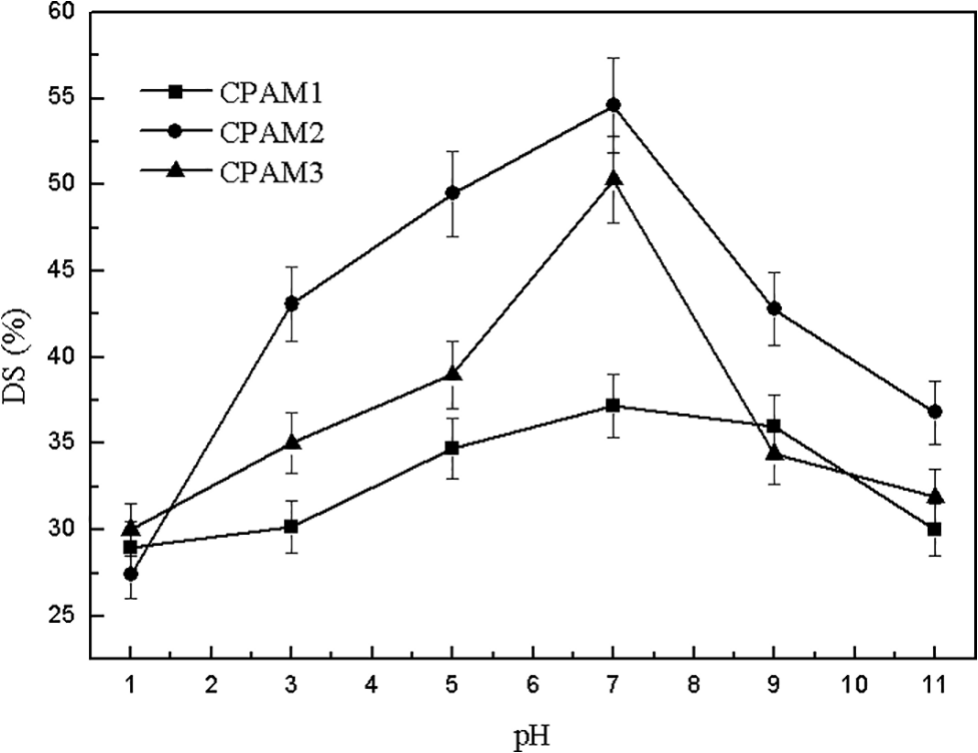
Effect of pH on DS.

The effect of pH on DS is shown in Fig 4, With increased pH, the DS values initially increased quickly, and then decreased rapidly. At pH 7, the maximum DS of CPAM1, CPAM2, and CPAM3 were 37.2%, 54.6%, and 50.3%, respectively. Sludge dewatering performances were much poorer under strong alkaline or acidic sludge than under neutral (weak acid/alkaline) condition. Therefore, neutral condition was suitable for sludge dewatering.

The turbid and yellow supernatant at pH 11 was probably caused by the hydrolysis of PAC, producing stable aluminum hydroxide particles that dispersed in the supernatant. The possible explanation for floating sludge at pH 1 was that the coagulant PAC in the sludge changed into Al^3+^ and further dissolved into the supernatant, leading to the reduction of the density of flocs.

As shown in Fig 4, the observed results show that the alum sludge after flocculation had good dewatering performance in neutral (weak acid/alkaline) condition. At low pH, the PAC in the sludge changed into Al^3+^, making the sludge particles tiny and reducing sludge dewatering performance; high pH also made the sludge particles fine, deteriorating sludge dewatering performance.

Verrelli [27] proposed that the precipitated inorganic coagulant in the sludge dominates dewatering behavior. The trends in the residual turbidity of supernatant and DS (Figs 3 and 4) both show considerable deterioration in sludge dewatering as the pH is decreased under pH 7 or goes the opposite above pH 7. The whole observed DS values substantiate this trend. The system pH has significant effects on the flocculation process: (1) it alters the overall solubility of the metal; (2) it alters the speed of the hydrolysis reaction; and (3) it favors the formation of precipitate phase. The pH also affected the extension degree of flocculant molecular chains in the solution, influencing the charge-neutralization ability and adsorption-bridging function, thus further affecting the flocculation performance and final sludge dewatering performance. According to reference [28], at about PH 6 the aluminum shows the lowest solubility. The better solubility accompanies by an increase of PH above 6, which is indicated to be a lower driving factor for precipitation, resulting to a more compact floc. A sludge consists of such flocs would perform a stronger dewaterability.

### Effect of dosage on SRF

**Fig 5:**
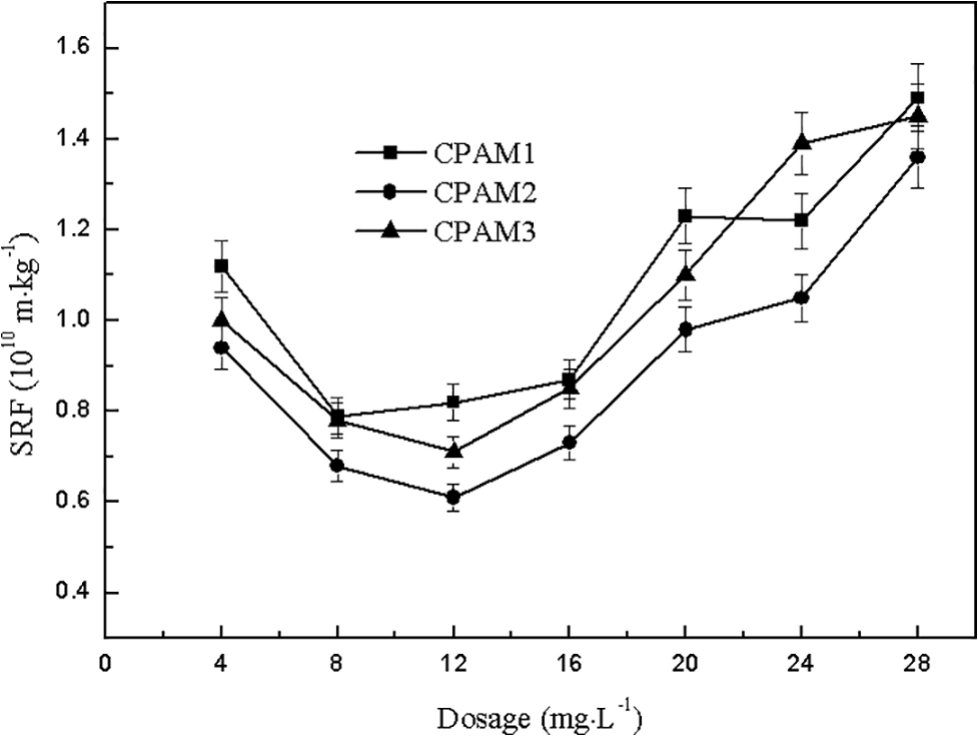
Effect of dosage on SRF.

The SRF of sludge was used to evaluate the degree of difficulty of sludge dewatering. The effect of cationic flocculant dosage on sludge specific resistance is shown in Fig 5 With increased dosage, the SRF of the sludge decreased initially, and then increased straight after 12 mg∙L^-1^ dosage. The minimum SRF of CPAM1, CPAM2, and CPAM3 were 0.82 × 10^10^, 0.61 × 10^10^, and 0.71 × 10^10^ m∙kg^-1^, respectively, obtained at 12 mg∙L^-1^ dosage. The original sludge SRF was 19.8 × 10^10^ m∙kg^-1^ without the addition of flocculant. The comparison shows that the SRF could be reduced and improve the dewatering performance significantly by addition of flocculant, making the sludge SRF decrease more than the observed magnitude. When the dosage was less than 20 mg∙L^-1^, the SRF of each flocculant was in the following order: CPAM1 > CPAM3 > CPAM2. As shown in Fig 5, CPAM2 with higher intrinsic viscosity had better flocculation performance and lower SRF compared with CPAM1. Meanwhile, between CPAM2 and CPAM3, the flocculant with higher cationic degree had lower SRF. Thus, the comparison indicated that high intrinsic viscosity or high cationic degree results in the reduction of the SRF and improvement of the sludge dewatering performance.

On the contrary, the SRF decreased initially, and then increased rapidly. The highly viscous flocculated alum of 28 mg∙L^-1^ accelerated the damage of filter paper during the vacuum filtration. as a whole, the value of SRF can reflect the difficulty of the sludge dewatering process[29]. The increase of SRF indicated that the pores in the bio-solids diminished and water transportation under high pressure suffered considerable obstruction. [30] Based on the combined DS and SRF variation trends for alum sludge, the polymer dosage of 12 mg∙L^-1^ was chosen as the optimal value for sludge flocculation, and the SRF of the corresponding alum was under 0.82 × 10^10^ m∙kg^-1^.

### Fractal dimension and floc size

Sludge particle size and particle size distribution were key factors that affect the performance of sludge dewatering. Sludge particles ranging from 1 μm to 100 μm were considered “supracolloidal” particles, and had the greatest effect on the dewaterability of sludge. When the concentration of the particles in this size range increased, dewaterability decreased. The density of “supracolloidal” particles was equal to or less than the density of water, leading to poor settling performance of super colloidal particles. Given that the particle size was close to the pore size of the filter, the great number of super colloidal particles in sludge brought about the unfavorable dewatering performance by readily penetrating through the filter [31–32]. Sludge floc formed after flocculation had a certain fractal structure, and the fractal dimension was used to investigate the structural characteristics of sludge flocs. Fractal dimension was closely related floc size, floc density, and floc porosity. The larger the floc fractal dimension is, the larger is the floc size and the greater is the precipitation rate.

**Fig 6:**
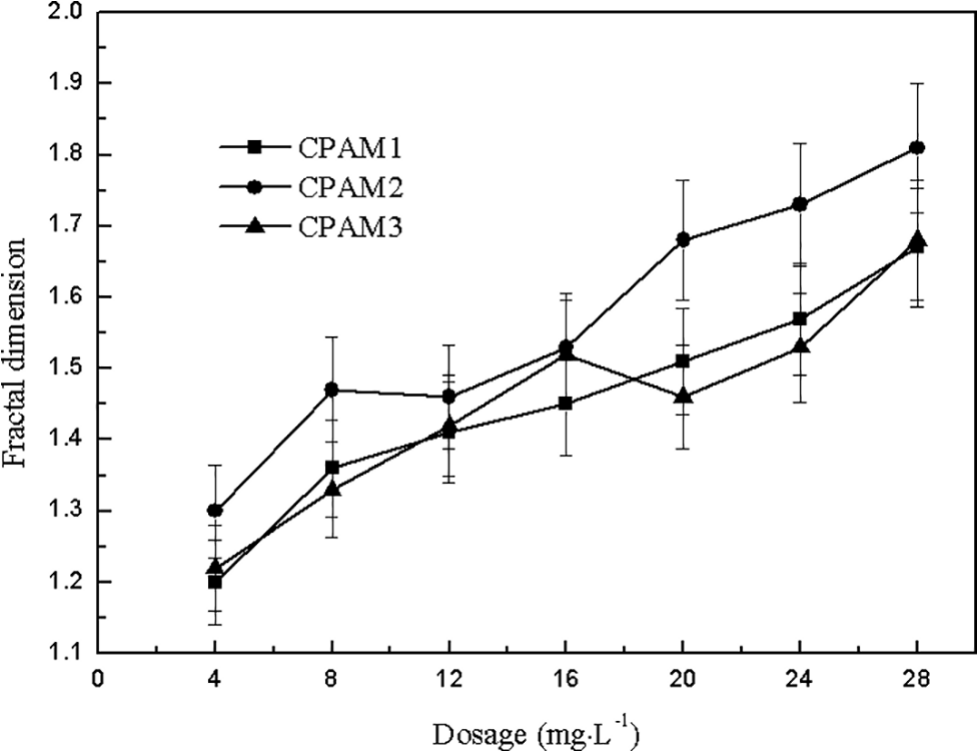
Effect of dosage on fractal dimension.

**Fig 7:**
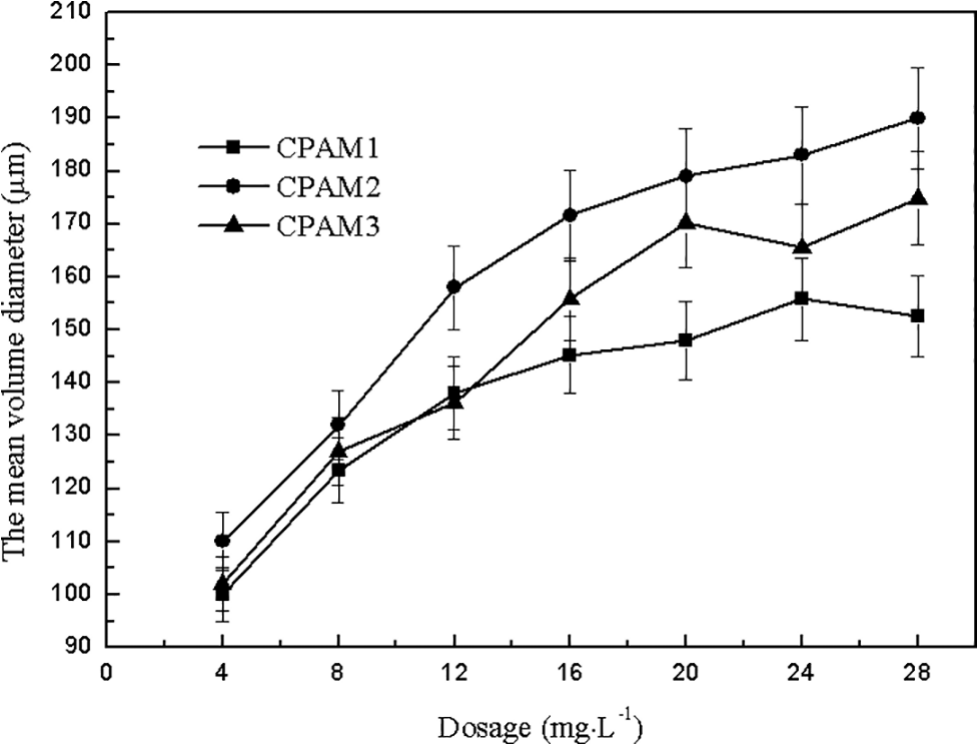
Effect of dosage on the mean volume diameter.

**Fig 8:**
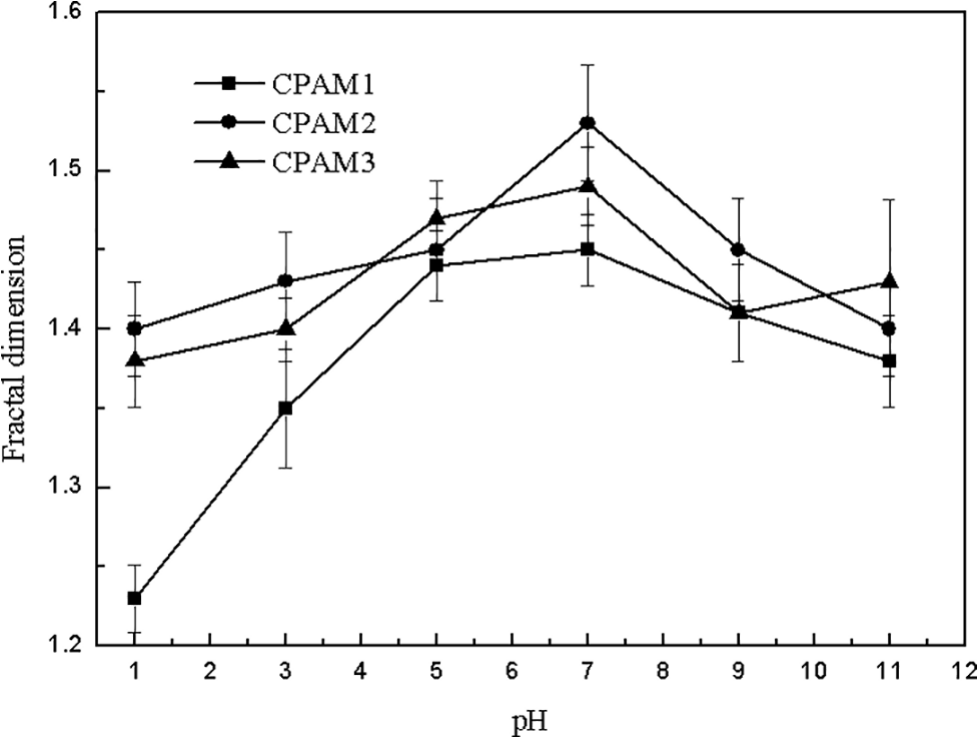
Effect of pH on fractal dimension.

The effect of dosage on fractal dimension and floc size is shown in Figs 6 and 7. As shown in Fig 6, the fractal dimension of sludge flocs increased with increased dosage. The fractal dimension of CPAM2 was greater than that of CPAM1 and CPAM3. Fig 7 and S1 Fig shows the effect of dosage on the floc size, which increased with increased dosage, and can be arranged in the following order: CPAM2 > CPAM3 > CPAM1. The evidence from Fig 7 and S1 Fig demonstrates that high floc size is associated with high flocculant dose. The trend of fractal dimension shown in Fig 6 is generally consistent with the trend of floc size shown in Fig 7 The effect of pH on fractal dimension is shown in Fig 8 The fractal dimension rapidly increased initially with increased pH and then slowly decreased. The fractal dimensions of sludge flocculated with different CPAM at different pH values were in the following order: CPAM2 > CPAM3 > CPAM1. At pH 7, the maximum fractal dimensions of CPAM1, CPAM2, and CPAM3 were 1.45, 1.53, and 1.49, respectively.

**Fig 9:**
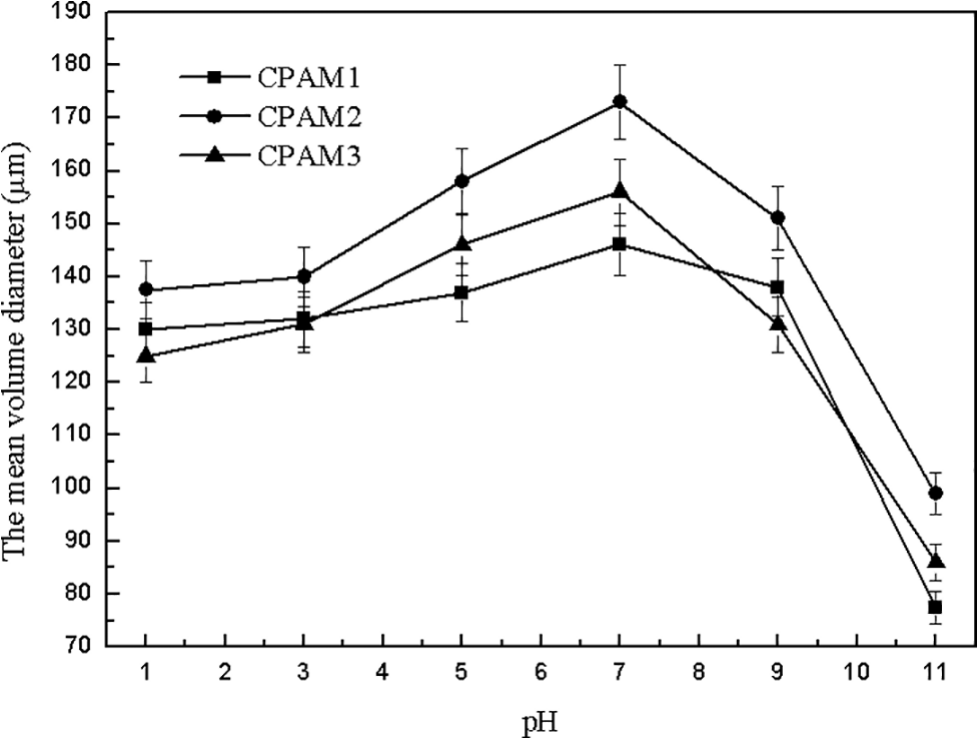
Effect of pH on the mean volume diameter.

As shown in Fig 9 and S2 Fig, the sludge floc size gradually increased with increased pH, and then decreased sharply, consistent with the trend shown in Fig 8 After flocculation at different pH values, the sludge floc sizes were generally in the following order: CPAM2 > CPAM3 > CPAM1. The maximum floc sizes of CPAM1, CPAM2, and CPAM3 obtained at pH 7 were 146, 173, and 156 μm, respectively. The experimental results show that the larger the fractal dimension is, the greater is the floc size, which is consistent with published literature [16]. The microscope photos of flocs were shown in the S1 Fig and S2 Fig, the observed results was consistent with Figs 7 and 9.

Fractal dimension was a quantitative measurement of floc structure and a description of how the original particles were organized within the floc interior [33]. Two points should be noted here: (1) fractal dimension at pH 7 was always larger than that at other pH levels, implying that the flocculation mechanism under neutral conditions was a combination of bridging and neutralization [34], and (2) the extremely loose floc structure obtained under alkaline conditions is mainly due to the repulsive forces between the negative charges of the adsorbed polymer and the sludge particles. The fractal dimension of flocs were calculated by image analysis of S1 and S2 Figs. As shown in Figs 6 and 7, the fractal dimension’s dependence on flocculant dosage is in accordance with the floc size results [35].

Table 3 shows the correlation between the fractal dimension and floc size after flocculation. The data in the table reveals a strong correlation between the fractal dimension and floc size after flocculation, where the correlation coefficients of CPAM1, CPAM2, and CPAM3 were all greater than 0.8 at the same dosage conditions, indicating a high positive correlation. The correlation coefficients of the three flocculant at the same pH were lower than 0.5853, especially for CPAM1, implying that a negative correlation exists between fractal dimension and floc size. A high fractal value indicates compact aggregates, whereas a low value corresponds to more ‘loose’ aggregates. Similarly, the floc strength was high for sludge flocs with high fractal dimension values, indicating that more dense flocs fragment less when exposed to shear. The floc Df rather than the floc size is the reflection of the internal floc structure. Nonetheless, the method of measuring Df is affected by the size of the flocs [36]. Large fractal dimensions signify more compact flocs, which are usually preferred in most water treatment situations to yield lower sludge volumes and for easier sedimentation.

**Table 3:** Correlation between fractal dimension and floc size.

Flocculants	Condition	Intercept	Slope	Linear Fit	Adj.R^2^
**CPAM1**	Same dosage	-38.30	121.06	y=121.06x-38.30	0.8419
	Same pH	57.14	50.55	y=50.55x+57.14	-0.2160
**CPAM2**	Same dosage	-76.80	151.29	y=151.29x-76.80	0.8115
	Same pH	-474.92	428.18	y=428.18x-474.92	0.5853
**CPAM3**	Same dosage	-94.41	164.52	y=164.52x-94.41	0.8079
	Same pH	-252.43	266.84	y=266.84x-252.43	0.02746

Previous literature [37] reported that the increase in sludge floc size after flocculation process can improve sludge dewatering performance; a low sludge SRF increases dewatered cake solid content, and a large sludge floc size after flocculation results in better sludge dewatering performance. This phenomenon results because small particles have a relatively large surface area with a large amount of adsorbed water. The increase in particle size and the decrease in specific surface area cause reduction in surface tension, thereby changing the sludge floc structure and reducing moisture absorption, resulting in improved sludge dewatering performance.

However, based on Figs 7 and 2, an opposite phenomenon occurred. The DS did not increase with the sludge floc size as reported in literature. This phenomenon is due to the increase in floc size caused by the increase of the flocculant dosage. Overdose effect can cause an increase in the viscosity of the liquid phase of sludge, and similar to a paper jam, increase the difficulty of filtration, thereby increasing the SRF and reducing cake solid content [38]. Meanwhile, overdose of flocculant can induce the formation of loose flocs, reduce floc steric effect, increase the amount of water contained inside the floc, decrease floc strength, and render the flocs weak and easily broken. The increase in fractal dimension would decrease the floc porosity and permeability, reducing the sludge’s settling properties [39].

### Zeta potential

To better discuss and explain the improvement of sludge dewatering performance by CPAM, knowing what the distribution of water in the activated sludge is necessary. Fortunately, the approach presented in latest literature about water distribution is relatively easy and accurate to apply [40–42].

Colin [43] established a distribution of water into different categories, namely, free water, bound water removable by moderate mechanical strain, bound water eliminated by maximal mechanical strain, and bound water not removable mechanically. Colin F [44] found that the flocculation process before sludge mechanical dewatering could change the distribution of water exit in the sludge flocs, increasing the content of free water. This result could be interpreted by two aspects: (1) the flocculant replaces water molecules binding on the sludge particle surface and (2) reduction of the hydration capacity of sludge particles. Chu [45] considered that when the dosage of flocculant sludge was less than the dosage of the isoelectric point, the improvement of sludge dewatering performance may be explained by the above discussion. However, the overdose effect would result in increasing the water content-combined sludge caused by the adsorption of water molecules in the molecule of the block organic flocculant.

**Fig 10:**
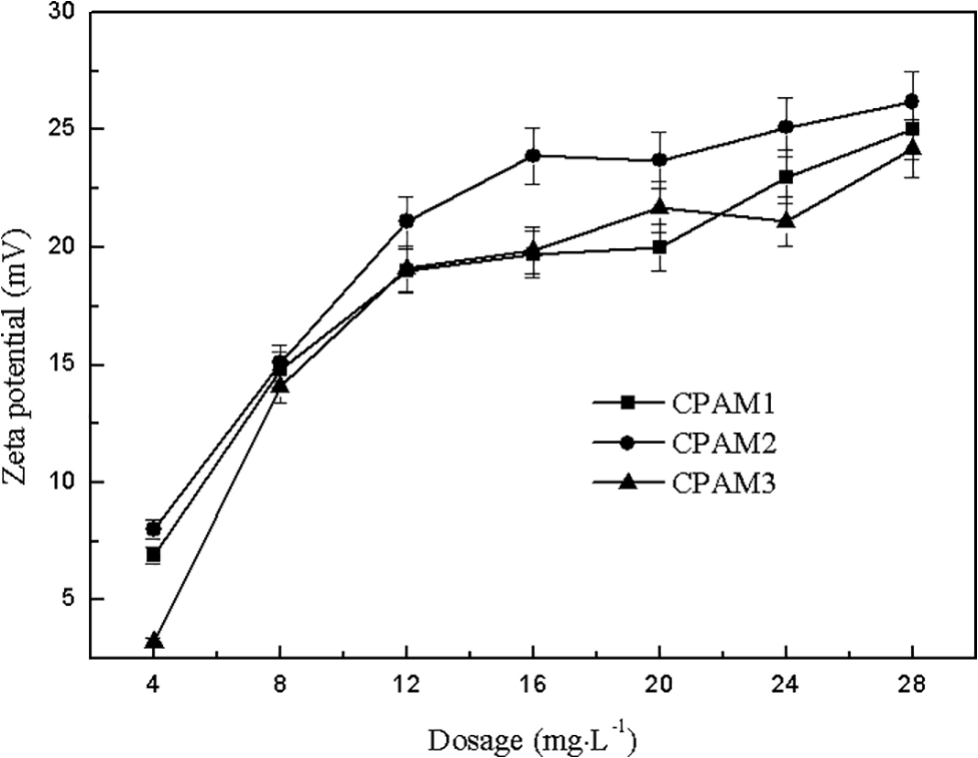
Effect of dosage on zeta potential.

As shown in Fig 10, with increased dosage, zeta potential was positive and increased continuously. The zeta potentials were in the following order: CPAM2 > CPAM1 > CPAM3, and this order was consistent with the order of cationic degree, indicating that the flocculant with higher cationic degree had better and stronger ability of charge neutralization. However, the zeta potential of sludge supernatant was –9.04 mV without addition of flocculant. When the flocculant was added, the negatively charged [Al(OH)_4_^-^] in the sludge was quickly neutralized, changing the zeta potential from negative to positive [46]. With the continuous increase in dosage, the sludge system no longer had a negative charge to be neutralized. However, the DS further increased mainly because of the adsorption bridging generated by flocculant chain. Flocculant polymer chains interacted with hydrophobic groups on the sludge surface by hydrogen bonding, van der Waals forces, and hydrophobic association, thus the adsorption bridging occurred. Hence, the main flocculation mechanism in flocculating alum sludge by cationic polyacrylamide was absorption and bridge effect.

**Fig 11:**
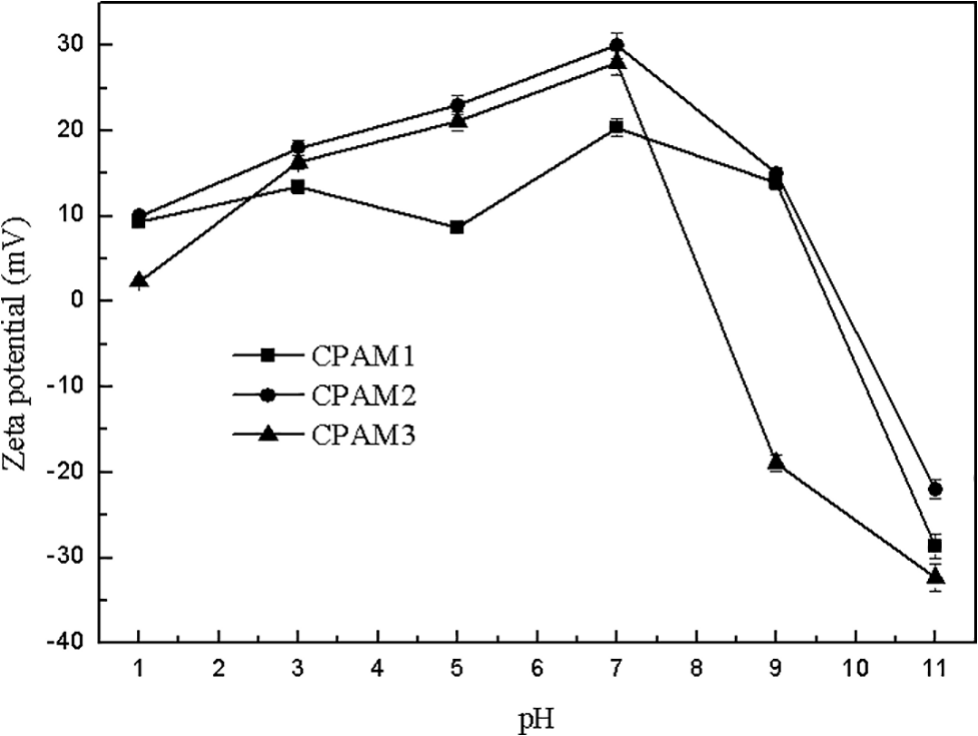
Effect of pH on zeta potential.

The effect of pH on the zeta potential is shown in Fig 11 With increased pH, zeta potential declined rapidly after the initial slow increase. When pH reached 7, the maximum zeta potentials of CPAM1, CPAM2, and CPAM3 were 20.4, 30.0, and 28.0 mV, respectively. Under acidic conditions, the high concentration of H^+^ in the sludge system made it positively charged and changed PAC into Al^3+^. The polymer chains are attached to the surface of particle at several points by adsorption to unattached segments that extend into the bulk of the liquid; the chain may loop back and attach to the same particle and other particles, eventually linking particles together to form flocs [47–48].

At high pH values, high concentration of OH^–^ in the sludge system concentration hydrolyzed the PAC into Al(OH)_3_ precipitate. The system sludge negative charge could not be neutralized by the fixed dosage of flocculant, so the zeta potential of the system was negative. The sludge particles were held by chain functional group and bridged together to form aggregates through charge neutralization and adsorption-bridging mechanisms. Therefore, the main flocculation mechanism was adsorption bridging under acidic condition, and charge neutralization and adsorption-bridging interaction under alkaline condition.

Table 4 shows correlation between floc size and zeta potential. As shown in Table 4, the correlation coefficients of CPAM1 and CPAM2 at the same pH or same dosage were greater than 0.92, whereas the correlation coefficient of CPAM3 was less than 0.9. In addition, the cationic degrees of CPAM1, CPAM2, and CPAM3 were 38%, 40%, and 21%, respectively, indicating that the flocculant with higher cationic degree had a larger correlation coefficient between floc size and zeta potential.

**Table 4:** Correlation between floc size and zeta potential.

Flocculants	Condition	Intercept	Slope	Linear Fit	Adj.R^2^
**CPAM1**	Same dosage	-22.44	0.30	y=0.30x-22.44	0.9496
	Same pH	-82.70	0.70	Y=0.70x-82.70	0.9711
**CPAM2**	Same dosage	-15.05	0.22	y=0.22-15.05	0.9706
	Same pH	-87.96	0.70	y=0.70-87.96	0.9281
**CPAM3**	Same dosage	-19.17	0.25	y=0.25-19.17	0.8645
	Same pH	-108.47	0.86	y=0.86-108.47	0.6875

## Conclusions

In this research, results of an experimental study on CPAMs as effective flocculation reagents for treatment of alum sludge from the drinking-water treatment plant were presented. During the process of flocculation and sludge dewatering, parameters that influence the sludge dewatering were experimentally tested. From the investigations, we came to the following conclusions:

The optimum conditions for the flocculation and sludge dewatering was at 16 mg∙L^-1^ flocculant dosage and pH 7, and under such conditions, the maximum DS was 54.6%. The minimum SRF of CPAM1, CPAM2, and CPAM3 were 0.82 × 10^10^, 0.61 × 10^10^, and 0.71 × 10^10^ m∙kg^-1^, respectively.

Floc size and floc fractal dimension increased with increased flocculant dosage. Correlation analysis revealed a strong positive correlation exists between fractal dimension and floc size after flocculation, where the correlation coefficients of CPAM1, CPAM2, and CPAM3 were greater than 0.8 at the same dosage condition. A low correlation between fractal dimension and floc size were found at the same pH, where the correlation coefficient was less than 0.6.

The maximum zeta potential was obtained at pH 7. A strong correlation exists between fractal dimension and zeta potential, and large correlation coefficient between floc size and zeta potential exists in the flocculant with high cationic degree. In addition, the main flocculation mechanism was adsorption bridging under the acidic condition, and charge neutralization and adsorption-bridging interaction under alkaline condition.

## Supporting Information

S1 Fig The microscope photos of flocs flocculated by CPAM2 at different dosage (40×). (DOCX)

S2 Fig The microscope photos of flocs flocculated by CPAM2 at different pH (40×). (DOCX)
