# Supplementary material for: Evaluation of Dewatering Performance and Fractal Characteristics of Alum Sludge
Source: PLoS One. 2015 Jun 29;10(6):e0130683. doi: 10.1371/journal.pone.0130683 (PMC4487249; doi:10.1371/journal.pone.0130683)
Supplement: S1 Fig — (DOCX) [file pone.0130683.s001.docx]

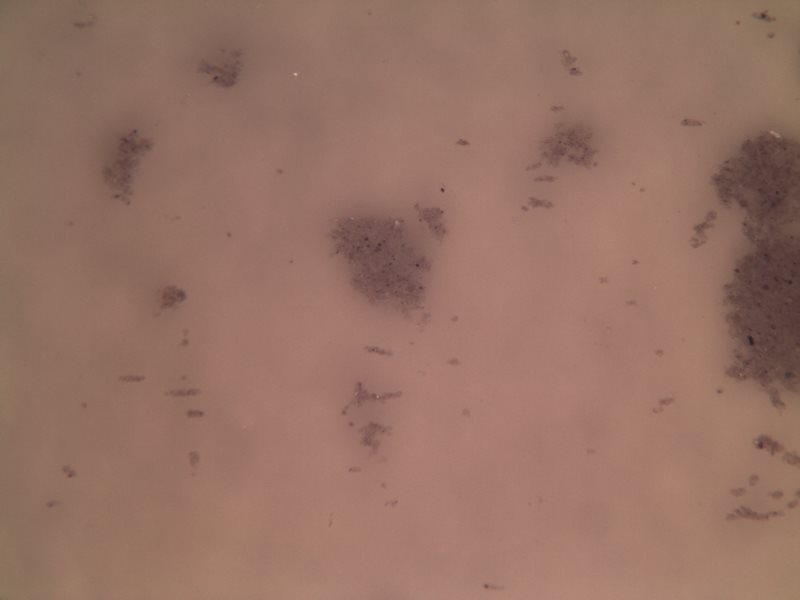

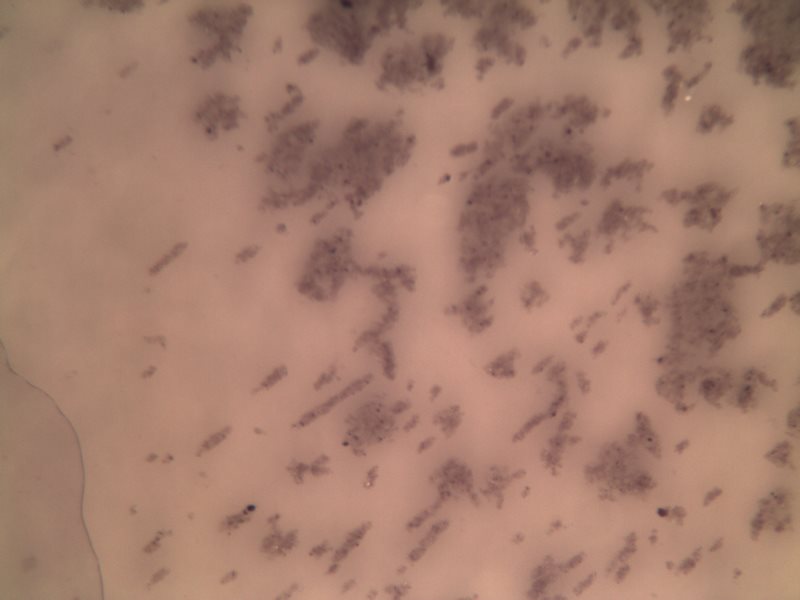


4 mg∙L^-1^ 8 mg∙L^-1^


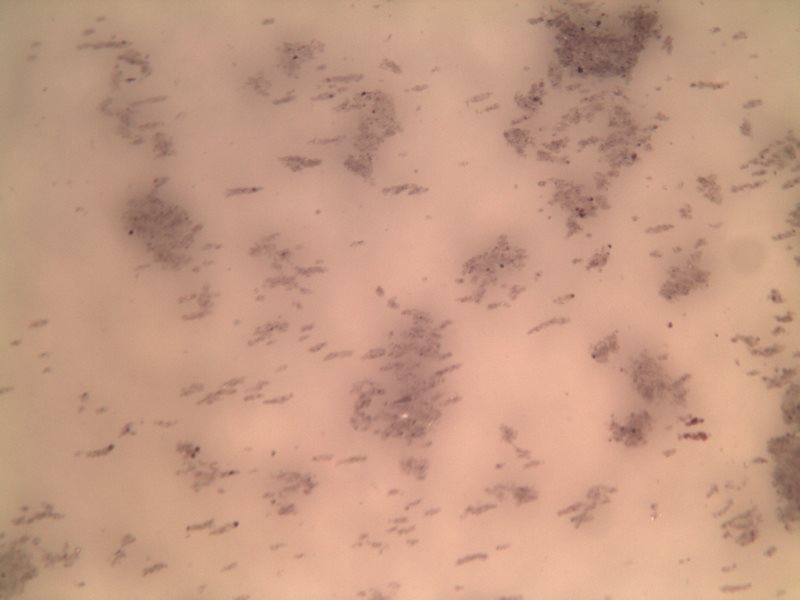

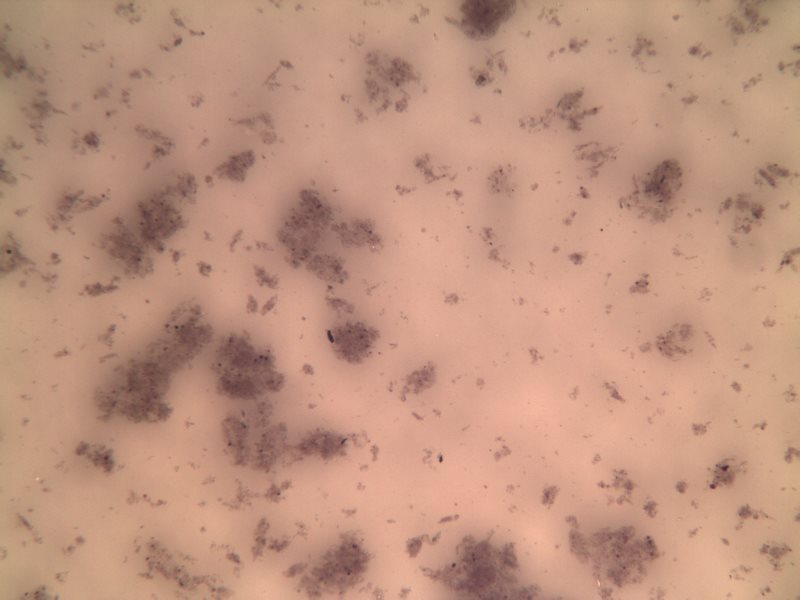


12 mg∙L^-1^ 16 mg∙L^-1^


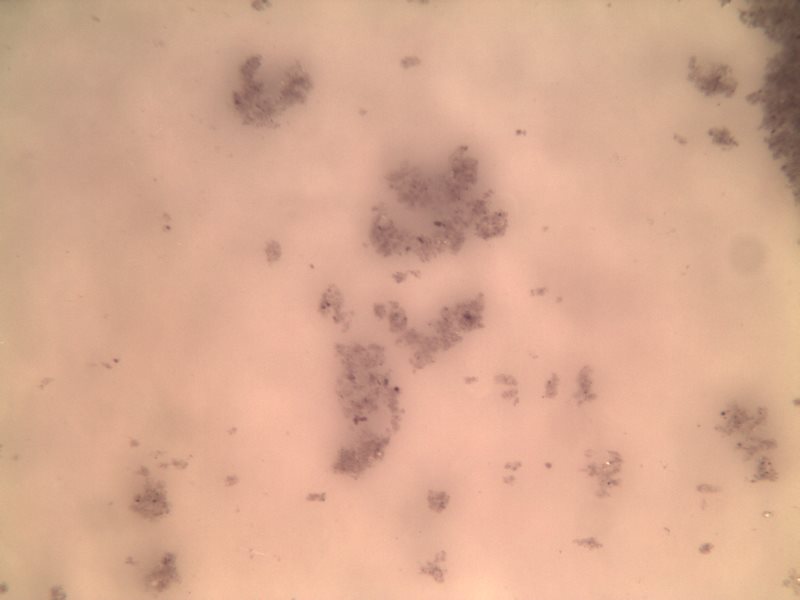

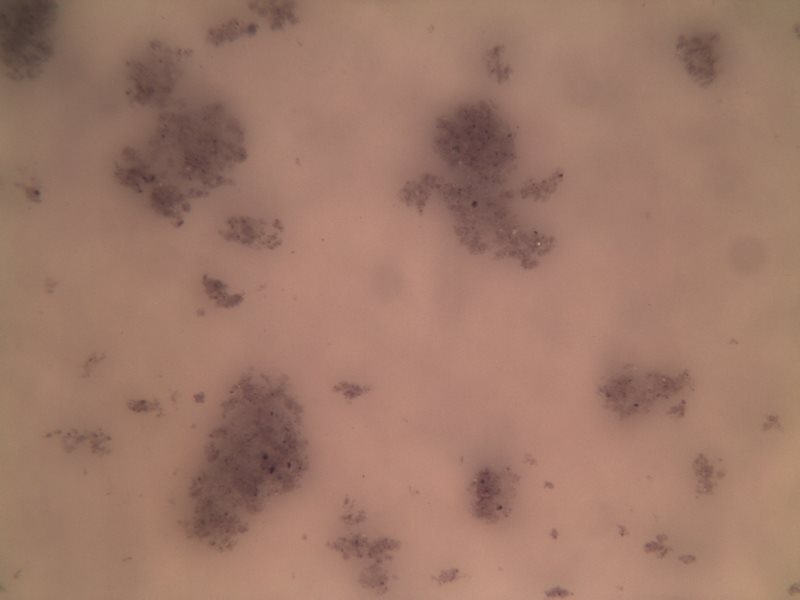


20 mg∙L^-1^ 24 mg∙L^-1^


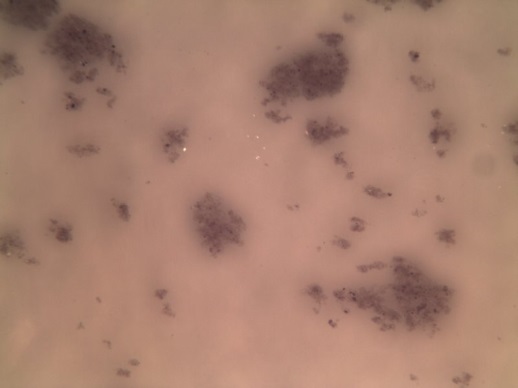


28 mg∙L^-1^

S1 Fig. the microscope photos of flocs flocculated by CPAM2 at different dosage (40×)
