# Supplementary figures and images for: Evaluation of Dewatering Performance and Fractal Characteristics of Alum Sludge
Source: PLoS One. 2015 Jun 29;10(6):e0130683. doi: 10.1371/journal.pone.0130683 (PMC4487249; doi:10.1371/journal.pone.0130683)

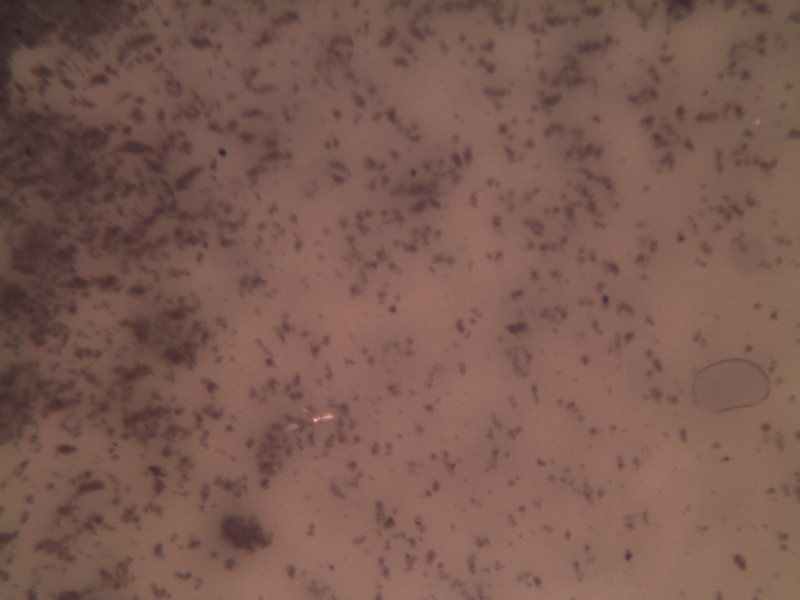

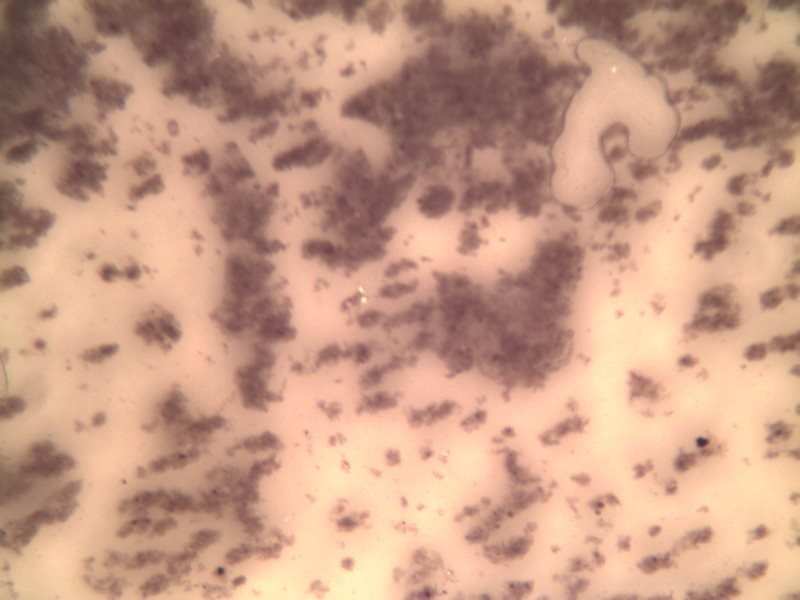


pH=1 pH=3


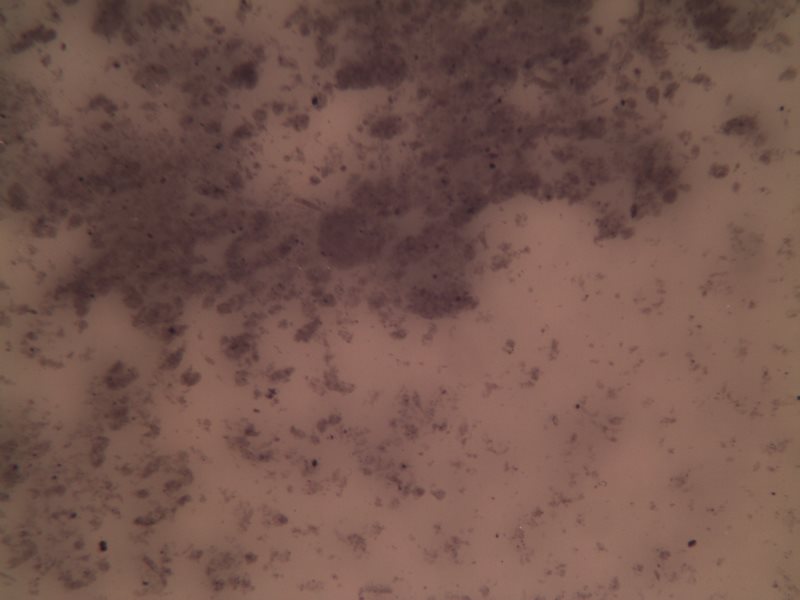

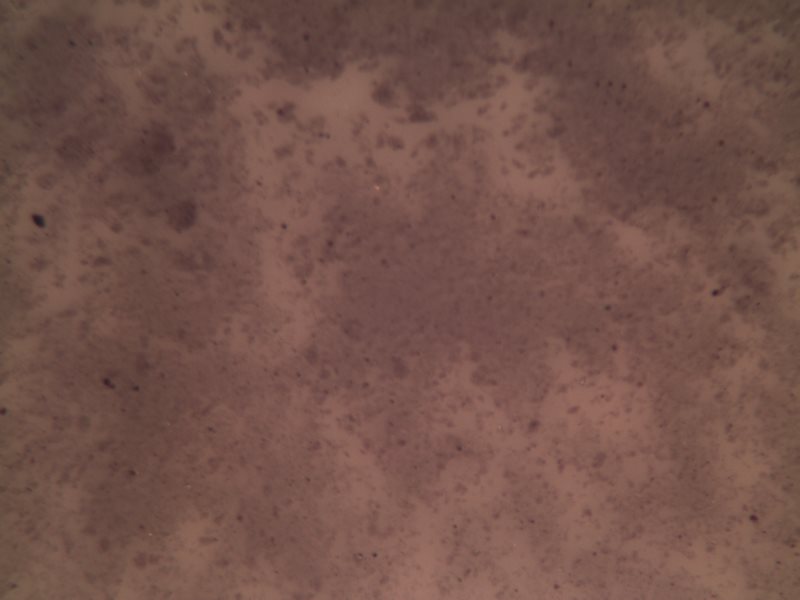


pH=5 pH=7


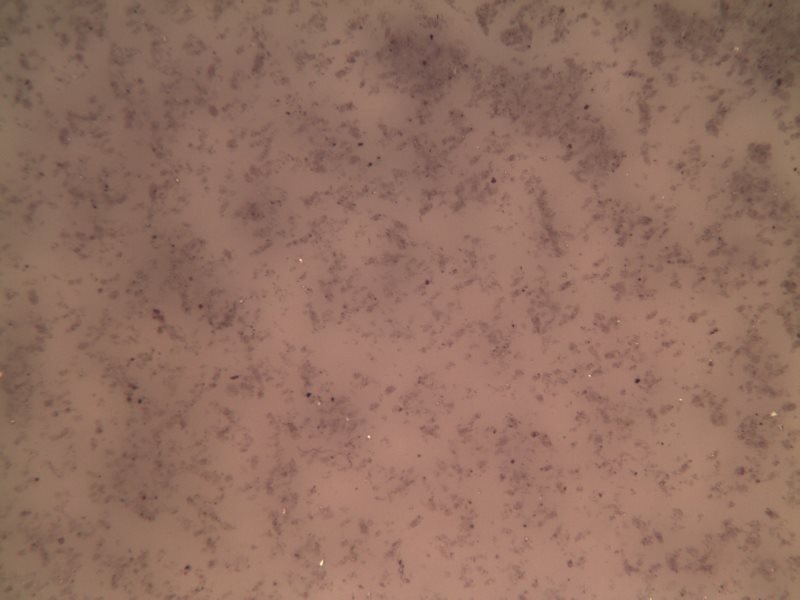

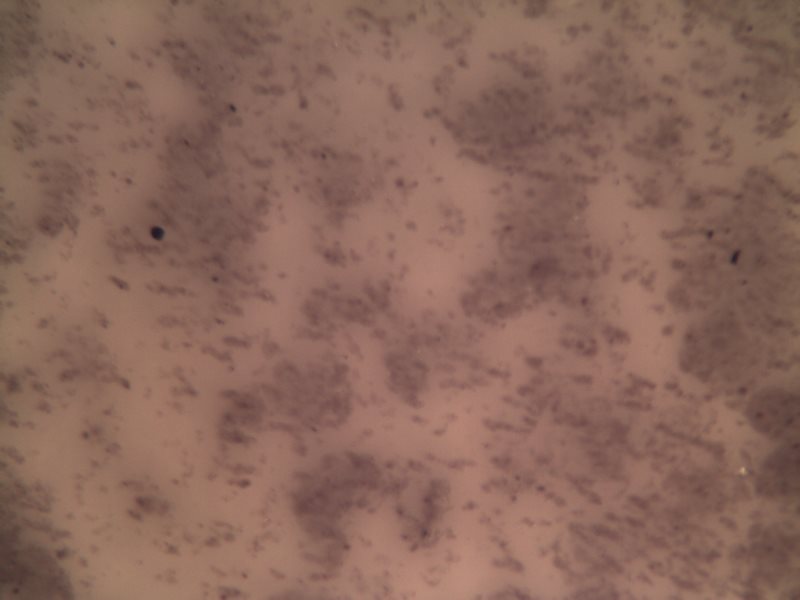


pH=9 pH=11

S2 Fig. the microscope photos of flocs flocculated by CPAM2 at different pH (40×)

Supplement: S2 Fig — (DOCX) [file pone.0130683.s002.docx]
